# Associations between two conceptualizations of materialism and subjective wellbeing in China: A meta-analysis of studies from 1998 to 2022

**DOI:** 10.3389/fpsyg.2022.982172

**Published:** 2022-09-23

**Authors:** Kaiji Zhou, Lin Lu, Liqun Hu, Yingzhao Wang

**Affiliations:** ^1^Department of Applied Social Sciences, The Hong Kong Polytechnic University, Hong Kong, Hong Kong SAR, China; ^2^School of Business Administration, Southwestern University of Finance and Economics, Chengdu, China; ^3^Sichuan Xiao Ping Executive Leadership Academy, Guangan, China

**Keywords:** materialistic values, extrinsic aspirations, subjective wellbeing, meta-analysis, Chinese samples

## Abstract

This meta-analysis examines the relationship between materialism (materialistic values and extrinsic aspirations) and subjective wellbeing in the Chinese population. Fifty-six relevant studies covering the period from 1998 to 2022 were included in the meta-analysis. Fifty-eight independent effect sizes from a total of 52,368 participants were obtained to calculate the mean effect sizes. Materialistic values correlated with significantly lower subjective wellbeing (*r* = −0.205), while the mean effect size for extrinsic aspirations was found to be not significant (*r* = −0.048). The effect sizes varied across different types of wellbeing outcomes (materialistic values: *r*s = −0.095 to −0.202; extrinsic aspirations: *r*s = 0.066 to −0.125). The associations were also moderated by certain demographic factors (age and gender), methodological factors (study design and scoring method), publication features (type of publication and publication year), and economic indicators (economic growth and wealth inequality). We discuss our limitations and the implications for future research.

## Introduction

Materialism, the orientation toward money and the acquisition of purchases that convey status as a means to gain success and personal wellbeing (Dittmar et al., 2014; Moldes and Ku, 2020), has surged worldwide since consumer culture emerged in the 20th century. As its market economy progressed, China has kept up with this trend over the past few decades, through the rise of its consumer culture marked by a raft of advertisements and the fashion industry that encourages the public to link happiness and success to wealth and material consumption (Zhou et al., 2017, 2018). This is evidenced by a survey by Ipsos covering twenty countries, which found that China has become the “most materialistic country.” (see Ipsos, 2013). Previous theoretical psychologists had warned that the pursuit of wealth cannot bring people psychological thriving, but even the opposite (Maslow, 1954; Kasser and Ryan, 1993, 1996). And past empirical studies had associated materialism with lower personal wellbeing (Chaplin and John, 2007; Roberts and Clement, 2007; Brdar et al., 2009; Martos and Kopp, 2012; Aruta, 2021). However, the empirical evidence from China is somewhat inconsistent: Both positive (e.g., Ling, 2013; Ye, 2017; Inseng Duh et al., 2021; Zhou, 2021) and negative correlations (e.g., Wang, 2012; Ma et al., 2020; Zheng, 2020; Zhou, 2021) between materialism and subjective wellbeing have been reported. It can be surmised, therefore, that materialism may not be invariably problematic in the Chinese context.

A comprehensive and quantitative analysis covering previous empirical studies is needed to gain a better understanding of the relationship between materialism and subjective wellbeing in China. An existing multi-national meta-analysis (Dittmar et al., 2014) included too few Chinese samples (China%_l_ = 1.6% vs. USA% = 49.6%) and did not report the results for Chinese samples. Given the unique socio-economic, cultural, and ideological characteristics of Chinese society (i.e., semi-market economy, Confucian values, and socialist ideology; see Chen, 1996; Yang and Stening, 2012, for details), it could be incorrect to assume materialism in China would exert impacts in the same way as western materialistic values. In addition, China has experienced dramatic socio-economic transformation and a rapid rise in consumer culture since its market economy transition marked by the implementation of the reform and opening up policy in 1978 (see Yang and Stening, 2016) and its entry into the World Trade Organization (WTO) in 2001 (Bao, 2008). It is interesting to observe how a rapid rise in consumer culture (reflected as materialism at the individual level) impacts people's wellbeing over this “golden age” in such a transforming society, providing a unique emic case for materialism research.

So far, the only existent meta-analysis based on Chinese samples (Zhou et al., 2018) was conducted 5 years ago and only considered a single conceptualization of materialism (materialistic values). Another important conceptualization of materialism, extrinsic aspirations (Kasser and Ryan, 1993, 1996), was missed. Therefore, we aim to gain a more general conclusion on how the materialism trend in Chinese society affects people's subjective wellbeing by quantitatively reviewing empirical studies using the two main conceptualizations of materialism. Furthermore, we aim to determine the sources of the inconsistencies in previous findings and respond to some theoretical debates by examining potential moderators.

### Materialism

Materialism is generally defined as an orientation toward money and the acquisition of purchases that convey status as a way to attain personal achievement and wellbeing (Richins and Dawson, 1992; Dittmar et al., 2014; Moldes and Ku, 2020). In line with this general definition, there are various specific conceptualizations in the materialism literature (e.g., Belk, 1986; Schwartz, 1992; Tang, 1992). In this meta-analysis, we include the two most widely used conceptualizations in China, materialistic values by Richins and Dawson (1992) and extrinsic aspirations by Kasser and Ryan (1993, 1996).

#### Materialistic values

The construct of materialistic values by Richins and Dawson (1992) is described as a set of beliefs that wealth and possessions play a central role in one's life (*Centrality*), material wealth is the source of happiness (*Happiness*), and wealth and possessions are symbols of success (*Success*; Richins and Dawson, 1992; Richins, 2004). The corresponding measure, the Materialistic Values Scale (MVS) composed of these three dimensions, has been widely used.

#### Extrinsic aspirations

Kasser and Ryan operationalized materialism as placing great importance on the goal of financial success or a broader range of extrinsic goals [fame, image, and financial success (Kasser and Ryan, 1993, 1996)]. The Aspiration Index (AI) has been widely used to measure extrinsic goals in materialism literature. Based on the AI, two different scoring methods have been adopted: (1) *absolute* scoring, simply reflecting respondents' ratings of the importance of extrinsic goals; (2) *relative* scoring, assessing the relative importance of extrinsic goals in comparison to intrinsic goals (self-growth, helping others, and health).

### Subjective wellbeing

Subjective wellbeing, in the general sense, is the best psychological function and experience of an individual, which can be measured by life satisfaction, positive affect, and negative affect (see Diener, 1994). The combination of the Satisfaction With Life Scale (SWLS; Diener et al., 1985) and the Positive Affect and Negative Affect Scale (PANAS; Watson et al., 1988) or a single measure containing the cognitive and affective aspects (e.g., Index of Wellbeing, IWB; Cummins, 1998) are commonly used for subjective wellbeing assessment. This wellbeing account is often described as *hedonic*. Another account, *eudemonic* wellbeing (Ryan and Deci, 2001), is often measured by the Psychological Wellbeing Scale (PWB; Ryff and Keyes, 1995; Ryff and Singer, 2010). This scale consists of six components: self-acceptance, self-growth, life goals, positive relationships, environmental mastery, and autonomy (Zhang and Zuo, 2007; Ryff and Singer, 2010).

### Relationship between materialism and subjective wellbeing

One of the famous explanations for the effects of materialism is derived from *Self-Determination Theory* (SDT), which suggests that pursuing materialistic goals is associated with poor satisfaction of the three basic psychological needs (competence, autonomy, and relatedness) considered essential for psychological thriving (Deci and Ryan, 2000). This mechanism has been empirically supported by past works (Niemiec et al., 2009; Qiu et al., 2012; Kasser et al., 2014; Wang et al., 2017; Li and Feng, 2018). Taking the universality of basic psychological needs in human beings as a prerequisite, the SDT account suggests that materialism is invariantly harmful to personal wellbeing across most demographic and contextual characteristics (see Deci and Ryan, 2000; Dittmar et al., 2014).

Another account, derived from a *person-environment value congruence* hypothesis (Sagiv and Schwartz, 2000), suggests that it is person-environment value fit (but not a specific value or goal) that determines personal wellbeing. In another word, one's materialistic values may enhance subjective wellbeing when the individual values match the dominant priorities of the surrounding environment (see Sagiv and Schwartz, 2000; Dittmar et al., 2014). To a certain extent, this hypothesis may explain the inconsistent findings regarding the correlations between materialism and personal wellbeing, which range from strong negative (e.g., Kasser and Ryan, 1996) to positive (e.g., Inseng Duh et al., 2021).

In recent years, some Chinese scholars, Li, Yang, and Guo, have pointed out that materialism could be a compensatory mechanism for psychological insecurity (see Kasser et al., 2004) and may be beneficial to self-esteem (Li et al., 2017). This mechanism may even improve personal wellbeing. Indeed, some empirical studies in China have shown the positive associations of pursuing materialistic goals with positive affective states and life satisfaction (e.g., Li, 2017; Ye, 2017).

Given the existent debates, we did not put forward a certain hypothesis on the relationship between materialism and subjective wellbeing in general but exploratively examined the direction and strength of the overall effect size. And we only proposed hypotheses for some moderator analyses.

### Moderators of the relationship between materialism and subjective wellbeing

The strength and direction of the correlation between materialism and subjective wellbeing may be affected by a number of factors. Prior meta-analyses found that the strength of the correlations depended on the sample features, publication characteristics, methodological factors, as well as economic and cultural factors (Dittmar et al., 2014; Zhou et al., 2018). In reference to the previous studies and based on the information available, we examined the following moderators:

#### Type of subjective wellbeing outcome

Although the meta-analysis conducted by Dittmar et al. (2014) did not find significant differences in effect size among the different outcome measures of subjective wellbeing, they did not compare the outcomes between eudemonic and hedonic approaches of measures (as noted above). We argue materialism, as the values aiming to gain pleasure from wealth and consumption, might not conflict with hedonic wellbeing, but conflict with eudemonic wellbeing which is more transcendence and spirituality related (Brdar et al., 2009; Zhou et al., 2017). Thus, materialism might be only negatively associated with eudemonic wellbeing but not hedonic wellbeing.

#### Scoring method of the AI (absolute vs. relative)

As noted above, the absolute scoring of the AI reflects respondents' ratings of the importance of extrinsic goals, while the relative scoring is able to assess the relative importance of extrinsic goals in comparison to intrinsic goals (by subtracting the average score of all AI goal items from the average score of the extrinsic goal items, and positive scores reflect a materialistic orientation). According to Kasser and Ryan (1993, 1996) and Grouzet et al. (2005), relative scoring provides a means-corrected measure of the relative centrality of extrinsic goals within the broader goal system of each person. Because any particular goal exists within a broader system of values and goals (Rokeach, 1973; Schwartz, 1992), the optimal assessment for extrinsic goals is capturing their relative importance within the goal system. To our knowledge, most Chinese studies only adopted absolute measures. It is necessary to reveal whether the research results were affected by the scorning method.

#### Study design (cross-sectional vs. longitudinal)

When the data is captured at the same time point, it may lead to common method bias and stronger correlation coefficients (Lu et al., 2022). Therefore, cross-sectional studies might yield larger effect sizes than longitudinal studies.

#### Sample features

Age and gender may also affect the size of the link between materialism and subjective wellbeing. In fact, a prior meta-analysis found a weaker negative correlation in adults than that in younger respondents (Zhou et al., 2018), indicating the moderating effect of age. We expected that the older the participants, the weaker the negative correlation between materialism and subjective wellbeing. The proportion of female respondents may also affect the size of the correlations. One study reported a weaker correlation among female samples (e.g., Ryan et al., 1999). However, Dittmar et al. (2014) found that a greater proportion of females in the samples could predict a stronger negative correlation between materialism and subjective wellbeing. Given these mixed results, we did not make a specific hypothesis but coded the proportion of females (if available) of each independent sample to examine the potential moderating effect of gender.

#### Publication characteristics

The correlation between materialism and subjective wellbeing might be moderated by publication year. As the years go by, China's “market-oriented transition” (Qian, 2000) is further deepened (Xin and Li, 2020). According to the person-environment congruence hypothesis, the enhanced consumer culture may provide individual materialistic values or goals with a supportive atmosphere that mitigates the negative effect of personal materialistic orientation on wellbeing. On the other hand, according to the consumer culture values impact model (Dittmar et al., 2013), individuals in a mass-consumer society are more frequently exposed to consumer culture cues (e.g., money, luxury goods, and perfect images attend to the advertising messages; Dittmar et al., 2013; Pellegrino et al., 2022), which will lead to more frequent self-discrepancies between one's current and ideal selves among materialistic individuals, thus strengthening the negative effects of materialistic orientation on self-evaluation, life satisfaction, and wellbeing. This meta-analysis examined the influence of publication year on the effect sizes to provide some evidence for these competing accounts.

Other publication characteristics, type of publication (published vs. unpublished) and language of publication (English vs. Chinese), may also affect the size of the correlations.

#### Economic characteristics

Given the relevance of economic conditions to materialism, this study examines the moderation roles of three economic indicators, per capita GDP indices, GDP percent growth, and the GINI coefficient. According to the goal-attainment hypothesis, a better economic condition (higher GDP and faster GDP growth) may mitigate the negative effects of materialism on individual life satisfaction and affective states because materialistic goals are more likely to be attained in an affluent society (Locke, 2007). However, according to the consumer culture values impact model (Dittmar, 2007; Dittmar et al., 2013), as mentioned above, the more frequent consumer culture messages in a wealthier and more commercialized society might cause more frequent self-discrepancies and a stronger link between materialism and poor personal wellbeing. This meta-analysis investigated the moderating effects of GDP and GDP growth to examine the competing hypotheses derived from goal-attainment and consumer culture values perspectives. The GINI coefficient reflects the extent of wealth inequality in society. Greater wealth inequality will likely make status differences, unequal opportunities, and social comparisons more salient to materialistic individuals (Dittmar et al., 2014). In turn, this causes greater negative self-discrepancies, dissatisfaction, and negative emotions. In other words, wealth inequality might make materialistic values and goals worse for individual wellbeing. Thus, we expected that the GINI coefficient would negatively moderate the relationship between materialism and subjective wellbeing.

### Summary

The relationship between materialism and subjective wellbeing is well-documented in the literature. Although existent theories and empirical studies have associated materialism with lower wellbeing, there are still debates and inconsistencies regarding the strength and stability of the relationship, which could be inevitable given the conceptual, methodological, and sociocultural differences among the existent studies. A worldwide meta-analysis has comprehensively reviewed the previous studies published in English and drawn general conclusions from an etic perspective, while China, as a potentially unique sample for materialism research, was almost omitted. Given the inconsistent empirical findings among the Chinese, comprehensive and quantitative analyses are needed to clarify the relationship between materialism and subjective wellbeing in China and to investigate whether the conclusions drawn at the global level are still applicable in the Chinese context.

This meta-analysis aims to examine the associations of the two main conceptualizations of materialism (materialistic values and extrinsic aspirations) with subjective wellbeing among Chinese samples, and we are interested in the potential difference in effect size between these two conceptualizations of materialism. We also examine the moderating effects of methodological factors (type of subjective wellbeing outcome measure, scoring method of the AI), sample features (type of participant, gender composition, and mean age), publication features (type of publication, publication year, and language of publication), and economic indicators (per capita GDP indices, GDP percent growth, and the GINI coefficient) to locate the sources of the inconsistent findings of previous studies and provide some direct or indirect evidence for the debates between different perspectives (i.e., consumer culture value impact perspective vs. person-environment value congruence and goal-attainment perspectives).

## Method

### Data collection

We used three search strategies to find relevant studies. First, a set of search terms including materialism, materialistic values, extrinsic goals, extrinsic aspirations, goal content, happiness, wellbeing, life satisfaction, China, and Chinese were used to search in online databases: EBSCO, Elsevier Science Direct, PsycINFO, ProQuest, Springer, SAGE, Wiley, Google Scholar, and OATD (Open Access to Theses and Dissertations). Studies published in Chinese were searched in Chinese online databases: CNKI and Baidu Scholar. Second, we carried out ancestor searches according to the reference lists of review articles and reports we obtained. Third, we contacted some scholars for unpublished papers or raw data sets. Data collection was up to May 2022.

### Inclusion criteria

**Figure 1:**
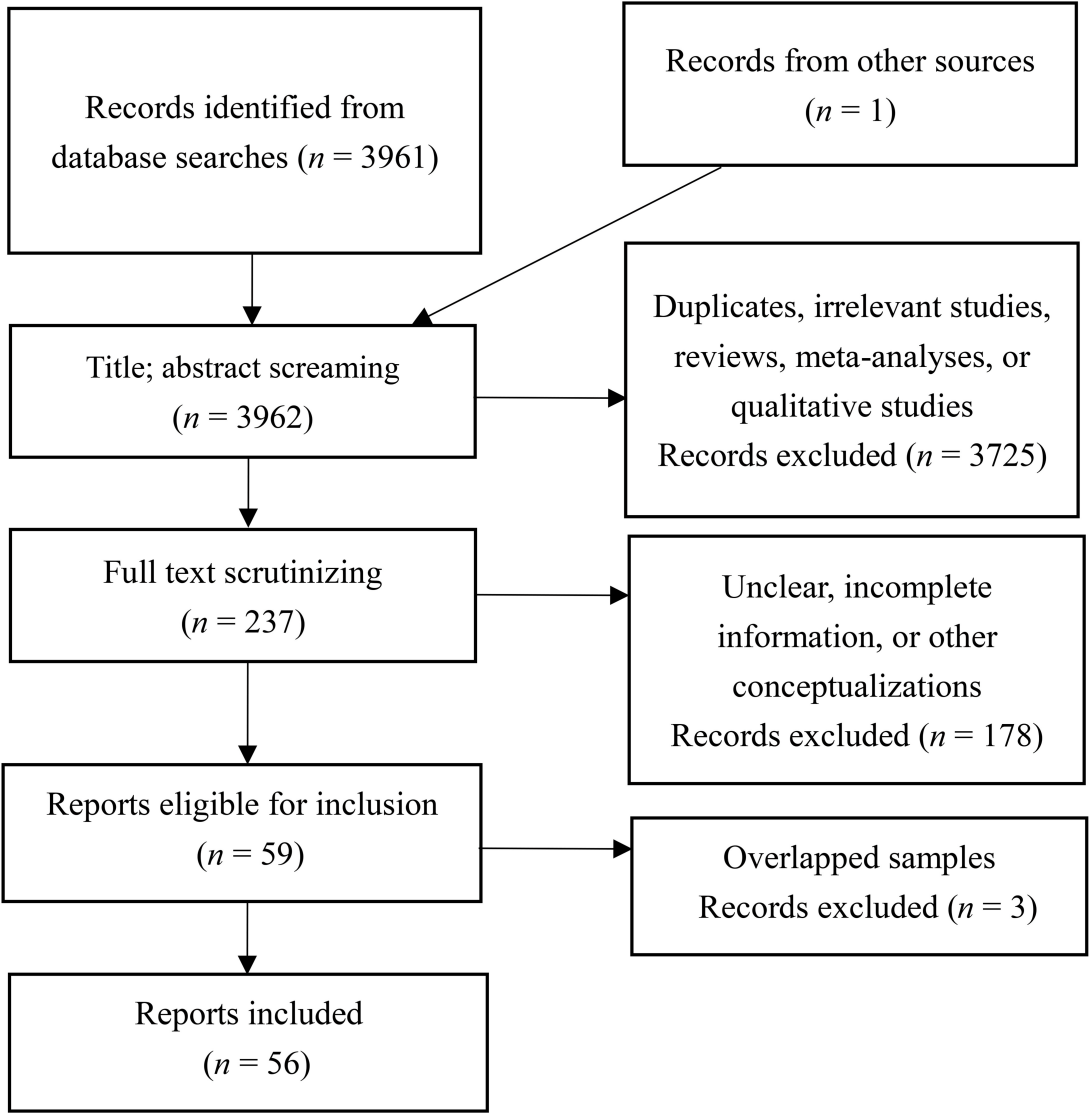
Literature search and inclusion (according to American Psychological Association Meta-Analysis Reporting Standards, MARS).

The studies included in this meta-analysis should meet eight major criteria: (1) must be empirical studies but not reviews or qualitative studies; (2) could be either correlational or longitudinal studies but not interventional or experimental studies that manipulated materialistic orientation; (3) materialism measures must be relevant to the construct of materialistic values (Richins and Dawson, 1992) or extrinsic aspiration (Kasser and Ryan, 1993, 1996); (4) outcome measures had to be relevant to the construct of subjective wellbeing (as described above in Introduction-Subjective wellbeing; see also Dittmar et al., 2014) but not specific emotions or symptoms (e.g., depression or anxiety); (5) the sample size should be clearly reported; (6) the participants should be Chinese; (7) Person's correlation coefficients *r* were provided, or *F, t*, β, and χ^2^ values that can be converted into *r* were provided; and (8) the samples of the included studies should be independent of each other. If multiple studies are retrieved from the same sample, only one of them would be included. The procedures for inclusion and exclusion are presented in Figure 1. Finally, a total of 56 studies were included in this study.

### Coding of studies

The studies were coded by study name, sample size, publication year, materialism measure, outcome measure, type of participant, type of publication, percent female, study design, mean age, and effect size. The results are shown in Table 1. The coding was based on independent samples; in the event that multiple effect sizes were produced by the same sample, only one of the effect sizes was selected (Lipsey and Wilson, 2008). When the multiple effect sizes produced by the same sample involved the moderators (e.g., longitudinal data or eudemonic wellbeing), they were coded separately and regarded as independent samples only in the moderator analyses. However, they were not included in the calculation of the overall mean effect size.

**Table 1:** A list of included studies.

**Reference**	***N***	**Materialism measure**	**Subjective wellbeing measure**	**Type of participants**	**Type of publication**	**% Female**	**Study design**	**Mean age**	***r* overall**	***r* LS**	***r* PA**	***r* NA**
Chen et al. (2014)	261	MVS-18	SWLS/PANAS	C	J	63.60	CS	23.07 ± 1.27		−0.350		
Inseng Duh et al. (2021)	207	MVS-8	OHS	C, Ad	J	51.69	CS	–	0.406			
Gao (2020)	244	MVS-13	IWB	C, Ad	J	77.87	CS	25.83 ± 7.01	−0.136	−0.097		
Gu and Qiu (2015)	398	MVS-15	SWLS+PANAS	C	J	63.56	CS	–	−0.210	−0.195	0.000	0.171
Gu et al. (2016)	418	MVS-15	SWLS+PANAS	C	J	63.39	CS	20.50 ± 1.50	−0.180	−0.200	−0.010	0.160
Guo and Yang (2021)	373	MVS-13	GWBS	C	J	68.55	CS		−0.240			
Guo (2014)	263	MVS-18	SHS	–	T	82.51	CS	21.29 ± 1.88	−0.138			
Guo et al. (2019)	575	MVS-C	IWB	C	J	49.4	CS	12.76	−0.200			
Huang (2014)	572	MVS-13	SWLS+PANAS	C	T	69.93	CS	–	−0.242	−0.187	−0.138	0.196
Jiang et al. (2012)	1,455	MVS-18	SWLS+PANAS	C	J	50.52	CS	21.3 ± 1.09	−0.320			
Jiang et al. (2016a)	764	MVS-13	SLSQA	C	J	60.21	CS	19.5		−0.170	−0.100	0.220
Jiang et al. (2016b) sample1	210	MVS-16	SWLS+PANAS	C	J	43.81	CS	19.93 ± 1.03	−0.330			
Jiang et al. (2016b) sample2	218	MVS-16	SWLS+PANAS	C	J	77.98	CS	19.68 ± 0.87	−0.240			
	⋆210						L		−0.210			
	⋆218						L		−0.240			
Jing (2017)	1,158	MVS-13	GWBS	C	T	62.69	CS	–	−0.266			
Li and Huang (2010)	253	MVS-13	IWB	Ad	J	–	CS	–	−0.021			
Li (2019)	698	MVS-13	GWBS	ES	T	48.71	CS	–	−0.247			
Li (2014)	918	MVS-9	SWLS /PANAS	–	J	52.40	CS	34		−0.090	−0.100	0.210
Lin (2011)	405	MVS-18	SWLS	C	T	58.02	CS	22	−0.130			
Lin (2020)	405	MVS-18	SWLS	C	J	–	CS	22	−0.130			
Yu (2018a)	3,563	MVS-13	SWLS	Ad	T	62.05	CS	–		−0.37		
Lu (2019)	179	MVS Self-developed	SWLS+PANAS	–	T	65.92	CS	–	−0.120	−0.170	−0.050	0.200
Luo (2018)	467	MVS-13	MSWBS-S	ES	T	56.51	CS	16.81	−0.274			
	⋆467		MSWBS-ST						−0.196			
Ma and Ding (2020)	386	MVS-13	SWLS	C	J	56.99	CS	–	−0.140			
Ma et al. (2020)	321	MVS-18	MHQ-S	C	J	54.52	CS	–	−0.226	−0.153	−0.119	0.214
	⋆321		MHQ-P						−0.182			
Ren et al. (2019)	537	MVS-13	IWB	Ad	J	100	CS	31.16 ± 3.04	−0.130	−0.096		
Sirgy et al. (1998)	187	MVS-7	SWLS	–	J	–	CS	32.88 ± 8.32	−0.165			
Wang et al. (2017)	565	MVS-18	SWLS	C	J	63.89	CS	19.34 ± 1.06	−0.270			
	⋆565						L		−0.190			
Wang (2012)	443	MVS-18	SWLS / PANAS	–	T	49.66	CS	–		−0.301	−0.067	0.207
Wang (2011)	402	MVS-15	MHQ-S	C	T	55.47	CS	–	−0.214	−0.178	−0.128	0.160
	⋆402		MHQ-P						−0.070			
Wen and Xu (2018)	380	MVS-13	SWLS+PANAS	C	J	54.21	CS	–	−0.021	−0.178	−0.074	0.182
	⋆380		PWBS					–	−0.250			
Xie et al. (2013)	701	MVS-18	SWLS,	ES	J	56.49	CS	–		−0.170	−0.100	0.150
	⋆701	MVS-18	PWBS	ES	J	56.49	CS	–	−0.190			
Xu (2013)	267	MVS-13	SWLS	C	J	52.81	CS	–	−0.118			
Yang (2018)	630	MVS-18	SWLS + PANAS	C	T	54.44	CS	–	−0.372			
Yao (2014)	525	MVS-10	ALSS + PANAS	ES	T	–	CS	–	−0.559			
Yu and Chen (2016)	946	MVS-18	SWLS,	C	J	54.02	CS	20.32 ± 0.15		−0.390	−0.340	0.320
	⋆946		PWBS						−0.271			
Yuan et al. (2012)	724	MVS-7	SWLS / PANAS	–	CP	49.59	CS	31.30 ± 11.6		−0.030	0.000	0.120
Zhao et al. (2019)	563	MVS-18	SWLS+SHS	Ad	J	41.74	CS	29.14 ± 2.37	−0.310	−0.220		
Zheng (2020)	547	MVS-15	SWLS+ PANAS	C	J	36.38	CS	–	−0.110	−0.110	−0.090	0.210
Zhou (2021)	3,981	MVS-5	SWLS	ES	D	46.09	CS	14.65 ± 1.11		−0.308		
Zhou et al. (2020)	225	MVS-13	MSLSS+ABS	C	J	63.72	CS	19.95	−0.070		−0.040	0.070
Du (2019)	575	AI-35 absolute	SWBS-C	C	J	53.57	CS	19.51 ± 1.03	−0.165			
Gatersleben et al. (2018)	961	Extrinsic goals	SWLS	–	J	–	CS	–	−0.250			
Ku (2015) sample 1	516	AI-12 relative	SWLS	ES	J	47.70	CS	12.94 ± 0.96		−0.180		
Ku (2015) sample 2	531	AI-12 relative	SWLS	ES	J	62.30	CS	16.57 ± 0.83		−0.200		
	⋆516						L			−0.170		
	⋆531						L			−0.360		
Lei and Huang (2015)	484	AI-35 absolute	SWLS / PANAS	C	J	50.62	CS	20.26 ± 1.31		−0.593	−0.672	0.389
Lekes et al. (2010)	515	AI-14 absolute	SCS+PANAS	ES	J	56.12	CS	15.5	0.130			
Qiu et al. (2012)	327	AI-18 absolute	SWLS+ PANAS	Ad	J	–	CS	23.61 ± 2.89	0.090			
Li and Feng (2018)	493	AI-35 absolute	IWB	–	J	47.70	CS	14.76 ± 1.23	−0.050			
Yu (2018b)	686	AI-35 absolute	SWLS+PANAS	ES	T	52.77	CS	–	−0.070			
Zheng et al. (2021)	1,567	1 item from the AI absolute	ABS	Md, C	J	–	CS	29.33 ± 6.00	−0.210			
	⋆1,567						L		−0.230			
Fan (2012)	194	AI-35 absolute	SWB-CC	Ad	T	42.46	CS	22.2 ± 4.2	0.010			
Li (2017)	15,870	AI-35 absolute	SWLS / PANAS	C, Ad	J	85.79	CS	26.9		0.047	0.032	0.080
	⋆15,870		OHS-eudemonic						0.183			
Ling (2013)	378	AI-35 absolute	SWLS,	Ad	J	100	CS	23.61 ± 2.80		0.202		
Wang (2008)	505	AI-35 absolute	SWLS / ABS	C	T	–	CS	–		−0.001	0.124	0.065
Ye (2017)	429	AI-18 absolute	SWLS+PANAS	C, Ad	T	60.14	CS	–	0.260	0.150	0.290	0.200
Yu et al. (2022)	796	AI-35 absolute	SWLS+PANAS	C	J	61.93	CS	19.2 ± 2.0	0.100			
Yu et al. (2015)	644	AI-35 absolute	SWLS /PANAS	C	J	33.85	CS	–		−0.050	0.040	0.090
Zhang (2019)	⋆135	AI-CE absolute	PWBS	Ad	T	39.26	CS	–	−0.086			
Zhou (2013)	563	AI-35 absolute	SWLS / PANAS	–	T	54.88	CS	–		0.173	0.004	−0.081

(1) Samples with a star (⋆) record effect sizes using longitudinal data or measures based on eudemonic approach, which were not included in the calculation of overall mean effect size to keep the independency of the effect sizes, and they were only included in subgroup analyses; (2) MVS, Materialistic Value Scales (Richins and Dawson, 1992; Li and Guo, 2009); MVS-C, Materialistic Value Scale for Children (Opree et al., 2011), AI = aspiration index (Kasser and Ryan, 1996; Tang et al., 2008), AI-CE, Aspiration Index for Chinese employee (Zhang, 2019); Extrinsic goals, extrinsic goal items (Kasser, 2005); a plus sign (+), the combination of the two measures of subjective wellbeing, a slash (/), using two measures of subjective wellbeing separately; SWLS, Satisfaction With Life Scale (Diener et al., 1985) and PANAS, Positive Affect Scale and Negative Affect Scale (Watson et al., 1988; Emmons, 1992; Qiu et al., 2008), ABS, Affect Balance Scale (Bradburn, 1969, 2015), SLSQA, School Life Satisfaction Questionnaire for Adolescents (Tao et al., 2005), ALSS, Adolescent Life Satisfaction Scale (Zhang et al., 2004), MSLSS, Multidimensional Students' Life Satisfaction Scale (Huebner and Gilman, 2002; Zhong et al., 2013), GWBS = General Wellbeing Schedule (Duan, 1996), SWBS-C, Subjective Wellbeing Scale for College Students (Ji and Li, 2006); SWB-CC, Subjective Wellbeing Scale for Chinese Citizens (Xing, 2003), PWBS, Psychological Wellbeing Scale (Ryff and Keyes, 1995), SCS, Self Concept Scale (Anderman, 2002). SHS, Subjective Happiness Scale (Lyubomirsky and Lepper, 1999), MHQ-S, Multiple Happiness Questionnaire-subjective wellbeing subscale; Miao, 2003), MHQ-P, Multiple Happiness Questionnaire-psychological wellbeing subscale; Miao, 2003), IWB, Index of Wellbeing (Campbell et al., 1976); OHS-eudemonic, Oxford Happiness Scale-eudemonic wellbeing subscale (Hills and Argyle, 2002), MSWBS-S, Multilayer College Students' Wellbeing Scale-subjective wellbeing subscale (Yang, 2015), MSWBS-ST, Multilayer College Students' Wellbeing-self-transcendent wellbeing subscale (Yang, 2015); (3) Ad, adults (graduated from college), C, college students, ES, elementary and/or secondary school students; (4) J, journal articles, T, thesis, CP, conference paper, D, data set; (5) CS, cross-sectional, L, longitudinal; (6) a dash (–), information is not available; (7) language of publication and country economic indicators were omitted to keep the size of the table.

We coded three macroeconomic indicators of the year in which a study was conducted: (1) per capita GDP indices (the adjusted per capita GDP which ensures comparability across different years); (2) GDP percent growth; (3) the GINI coefficient (which represents wealth inequality). The data of the economic indicators were obtained from NBSPRC (National Bureau of Statistics of the People's Republic of China), Statistical Yearbook of China, and Research Office of the State Council. We recorded data on each for the year in which the study was conducted. For studies that did not report the year of data collection, we used the method of cross-temporal meta-analysis, subtracting 2 years from the year of publication as the year in which the study was conducted (Oliver and Hyde, 1993; Twenge et al., 2010).

### Data analysis

Comprehensive meta-analysis 3.0 (CMA 3.0) software was adopted for data analysis. CMA 3.0 uses Hedges-Olkin method (Shadish and Haddock, 2009) for correlational meta-analysis. Random-effects models were used to calculate the mean effect sizes (Borenstein et al., 2009). The heterogeneity test (*Q* test) was used to evaluate the variance among the independent samples. Funnel Plot and Fail-safe Number (*Nfs*) were used to assess the publication bias.

## Results

### Data set description

In total, we included 56 studies (published from 1998 to 2022) with 126 effect sizes in this meta-analysis. Fifty-eight independent effect sizes (40 for materialistic values and 18 for extrinsic aspirations) were taken to calculate the overall effect sizes; the rest of the effect sizes were only used in subgroup analyses with the independence of the effect sizes in each subgroup guaranteed. The summary of the study characteristics is shown in Table 2.

**Table 2:** Study characteristic.

**Characteristic**	**Materialistic values**		**Extrinsic aspirations**	
	***k***	***n***	***k***	***n***
Type of participant				
Adults, college students mixed	2	451	2	16,299
Adults	4	4,916	3	899
College students	23	11,881	5	3,004
Elementary and/ or secondary school students	5	6,372	4	2,248
Publication year				
Median	2016.5		2015	
Range	1998–2021		2008–2022	
1998~2008	1	187	1	378
2009~2019	30	19,458	15	30,203
2020~2022	9	6,689	2	1,073
Type of publication				
Journal article	26	12,324	13	23,657
Thesis	12	9,305	5	2,377
Conference paper	1	724		
Data set	1	3,981		
Language of publication				
English	9	3,522	6	3,210
Chinese	30	18,831	12	22,824
Design				
Cross-sectional	40	26,334	18	26,034
Longitudinal	⋆3	993	⋆3	2,614
AL scoring method				
Absolute			15	24,026
Relative			2	1,047
% Female				
Median	56.49 (*k* = 36)		54.23 (*k* = 14)	
Range	36.38~100%		33.85~100%	
Mean age				
Median	21.29 (*k* = 21)		19.89 (*k* = 12)	
Range	12.76~34.00		12.94~29.33	
Sample size				
Median	430.5		516	
Range	179~3,981		194~15,870	

*k* values with a star (⋆) record effect sizes yielded by longitudinal data, which were not be included in main effect analyses and were only be taken in moderator analyses (subgroup analyses); to compare the college student samples with other adult samples, the subgroup of “Adults” in this study did not include the college students, and the “Adults, college students mixed” subgroup was used to present the samples containing both college students and other adults; because elementary school students and secondary school students were often mixed in samples, these two types of participants were included in the same subgroup.

### Publication bias evaluation

**Figure 2:**
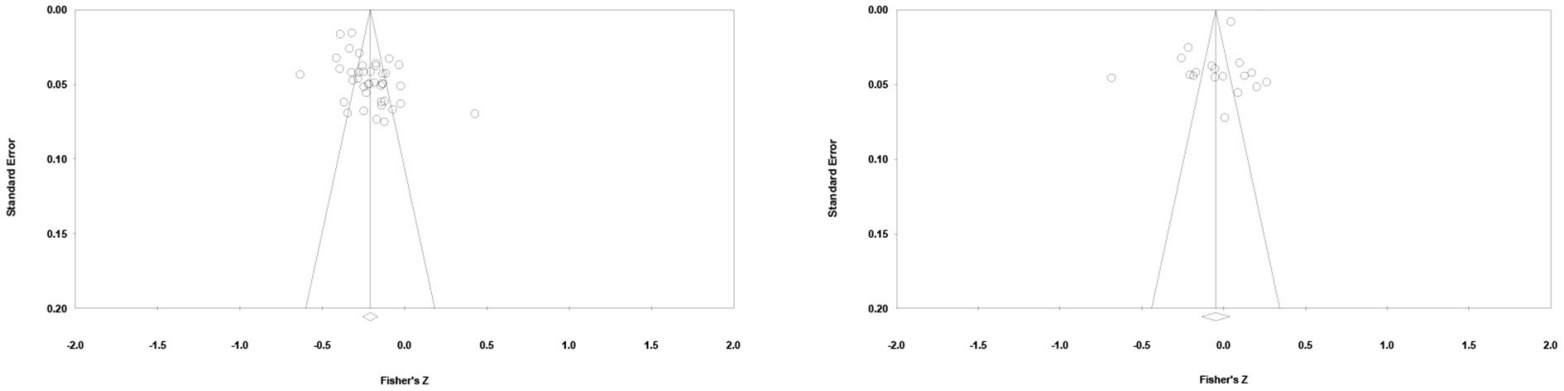
Funnel plots; materialistic values (left), extrinsic aspirations (right).

The publication bias test was carried out through the funnel plot and the *Nfs*. The results are shown in Figure 2. It can be seen from Figure 2 that the effect sizes yielded by most of the dependent samples are concentrated on the top of the funnels and are evenly distributed on both sides, indicating a small possibility of publication bias. The *Nfs* for materialistic values = 11,858, >5*k*+10, indicating that the mean effect size for materialistic values was not affected by publication bias. The *Nfs* for extrinsic aspirations = 100 (reached the threshold, 5k+10). Despite the relatively small *Nfs*, the non-significant mean effect size for extrinsic aspirations was not likely to be affected by publication bias.

### Main effect analyses

As shown in Table 3. There was significant heterogeneity among studies on both materialistic values (*Q* = 473.512, *p* < 0.001) and extrinsic aspirations (*Q* = 535.366, *p* < 0.001), indicating that random-effects models are suitable for the overall effect size calculation. The association between materialistic values and subjective wellbeing was significant (*r* = −0.205, *p* < 0.001), while there was no significant correlation between extrinsic aspirations and subjective wellbeing (*r* = −0.048, *p* = 0.268). The difference in effect size between materialistic values and extrinsic aspirations was significant (*Q*_B_ = 10.596, *p* = 0.001).

**Table 3:** Main effects of two conceptualizations of materialism.

				**95%** ***CI*** **for** ***r***			
**Materialism**	***k***	***N***	***r***	***LL***	***UL***	***Z***	***Q***
Materialistic values	40	26,334	−0.205	−0.247	−0.163	−9.287^***^	473.512^***^
Extrinsic aspirations	18	26,058	−0.048	−0.133	0.037	−1.106	535.366^***^
*Q*_*B*_ = 10.596^**^							

*LL*, lower limit; *UL*, upper limit;

** *p* < 0.01,

*** *p* < 0.001;

*Q*_B_, between group *Q* value.

### Moderator analyses

#### Type of subjective wellbeing outcome

As shown in Table 4. The relationship between materialistic values and subjective wellbeing was moderated significantly by type of subjective wellbeing outcome (*Q*_B_ = 12.363, *p* = 0.015). The negative correlation was significant across different types of outcomes, and the effect size for positive affect (*r* = −0.095, *p* < 0.001) was weaker than others (*r*s = −0.192 to −0.202, *p*s < 0.001).

**Table 4:** Moderation by type of subjective wellbeing outcome.

				**95%** ***CI*** **for** ***r***			
**Outcome variable**	***k***	***n***	***r***	***LL***	***UL***	***Z***	***Q*_B_**
Materialistic values							12.363^*^
Life satisfaction + positive affect, negative affect	17	7,806	−0.202	−0.251	−0.152	−7.739^***^	
Life satisfaction	21	17,087	−0.197	−0.248	−0.144	−7.169^***^	
Positive affect	15	7,938	−0.095	−0.147	−0.042	−3.494^***^	
Negative affect (reversed)	15	7,938	−0.192	−0.223	−0.160	−11.715^***^	
Eudemonic wellbeing	6	3,217	−0.198	−0.253	−0.141	−6.723^***^	
Extrinsic aspirations							6.630
Life satisfaction + positive affect, negative affect	5	2,755	0.066	−0.051	0.180	1.107	
Life satisfaction	9	19,920	−0.060	−0.199	0.082	−0.826	
Positive affect	6	18,495	−0.052	−0.271	0.171	−0.454	
Negative affect (reversed)	6	18,495	−0.125	−0.223	−0.025	−2.450^*^	
Eudemonic wellbeing	2	16,005	0.063	−0.199	0.317	0.469	

*LL*, lower limit; *UL*, upper limit;

* *p* < 0.05,

*** *p* < 0.001;

*Q*_B_, between group *Q* value.

The association between extrinsic aspirations and subjective wellbeing could not be moderated by type of outcome. However, the effect size for negative affect (reversed) was the only significant one (*r* = −0.125, *p* = 0.014).

#### Type of participant

The results of the moderating analyses on type of participant are shown in Table 5. For materialistic values, although between-group variation was not significant, the negative correlation was slightly stronger in elementary and middle school students (*r* = −0.318, *p* < 0.001) than in older age groups (e.g., adults *r* = −0.218, *p* = 0.007). We further examined the moderation effect of mean age by meta-regression.

**Table 5:** Type of participant, study design, type of publication, language of publication, and AI scoring method as moderators.

				**95%** ***CI*** **for** ***r***			
**Moderator**	***k***	***n***	***r***	***LL***	***UL***	***Z***	***Q*_B_**
**Materialistic values**							
Type of participant							4.409
Adults, college students mixed	2	451	0.145	−0.388	0.606	0.516	
Adults	4	4,916	−0.218	−0.364	−0.061	−2.711^**^	
College students	23	11,881	−0.219	−0.260	−0.178	−10.112^***^	
Elementary and/or secondary school students	5	6,372	−0.318	−0.424	−0.204	−5.278^***^	
Study design							0.000
Cross-sectional	40	26,334	−0.205	−0.247	−0.163	−9.287^***^	
Longitudinal	3	993	−0.205	−0.264	−0.145	−6.530^***^	
Type of publication							5.052^*^
Published	26	12,324	−0.172	−0.223	−0.119	−6.293^***^	
Unpublished	14	14,010	−0.255	−0.321	−0.186	−7.054^***^	
Language of publication							
English	9	3,522	−0.175	−0.294	−0.051	−2.749^**^	0.267
Chinese	30	18,831	−0.209	−0.257	−0.161	−8.257^***^	
**Extrinsic aspirations**							
Type of participant							7.636*^*ms*^*
Adults, college students mixed	2	16,299	0.150	−0.063	0.350	1.383	
Adults	3	899	0.109	0.001	0.215	1.973^*^	
College students	5	3,004	−0.158	−0.393	0.096	−1.220	
Elementary and/or secondary school students	4	2,248	−0.081	−0.222	0.063	−1.102	
Study design							9.543^**^
Cross-sectional	18	26,034	−0.048	−0.133	0.037	−1.106	
Longitudinal	3	2,614	−0.254	−0.348	−0.155	−4.927^***^	
AI scoring method							9.907^**^
Absolute	15	24,026	−0.015	−0.107	0.077	−0.316	
Relative	2	1,047	−0.190	−0.248	−0.131	−6.211^***^	
Type of publication							4.025^*^
Published	13	23,657	−0.095	−0.199	0.012	−1.741*^*ms*^*	
Unpublished	5	2,377	0.076	−0.052	0.203	1.163	
Language of publication							0.669
English	6	3,210	−0.095	−0.216	0.083	−0.463	
Chinese	12	22,824	−0.026	−0.134	0.083	−0.464	

To compare the college student samples with other adult samples, the subgroup of “Adults” in this study did not include the college students, and the “Adults, college students mixed” subgroup was used to present the samples containing both college students and other adults; because elementary school students and secondary school students were often mixed in samples, these two types of participants were included in a subgroup; *Q*_B_, between-group *Q* value; ^ms^, marginal significance; *LL*, lower limit; *UL*, upper limit;

* *p* < 0.05,

** *p* < 0.01.

*** , *p* < 0.001.

For extrinsic aspirations, the difference among different types of participants reached marginal significance (*Q*_B_ = 7.636, *p* = 0.054). There was a positive and significant correlation between extrinsic aspirations and subjective wellbeing (*r* = 0.109, *p* = 0.048) among adults, and the effect sizes were not significant in other subgroups.

#### Study design

As shown in Table 5, the strength of the association between extrinsic aspirations and subjective wellbeing depended on study design (*Q*_B_ = 9.543, *p* = 0.002). The longitudinal data yielded a larger effect size (*r* = −0.254, *p* < 0.001) than cross-sectional studies (*r* = −0.048, *p* > 0.05). The moderating effect of study design on the relationship between materialistic values and subjective wellbeing was not significant.

#### Type of publication and language of publication

The association between materialistic values and subjective wellbeing was moderated by type of publication (Table 5), the effect size for published articles (*r* = −0.172, *p* < 0.001) was smaller than unpublished sources (*r* = −0.255, *p* < 0.001; *Q*_B_ = 5.052, *p* = 0.025). For extrinsic aspirations, the difference between published sources and unpublished sources was significant (*Q*_B_ = 4.025, *p* = 0.045). The effect size for published articles reached marginal significance (*r* = −0.095, *p* = 0.082), while the result of unpublished sources was not significant. The moderating effect of language of publication (English vs. Chinese) was not significant.

#### Scoring method of the AI

As shown in Table 5. The moderating effect of scoring method was significant (*Q*_B_ = 9.907, *p* = 0.002). The negative correlation was only significant in the group of relative scoring method (*r* = −0.190, *p* < 0.001), but not significant in the absolute scoring group.

#### Mean age, percent female, and publication year

**Figure 3:**
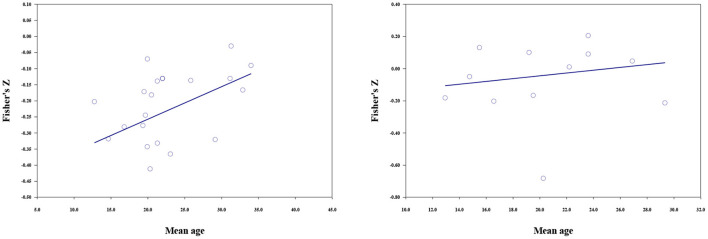
The moderating effect of mean age; materialistic values (left), extrinsic aspirations (right).

The results of meta-regressions are shown in Table 6. For materialistic values, the moderating effect of mean age was significant (*b* = 0.010, *p* < 0.001), the higher the mean age, the weaker the negative correlations (Figure 3). Publication year could moderate the relationship between materialistic values and subjective wellbeing (*b* = −0.005, *p* = 0.009). Studies published in more recent years reported stronger negative correlations (**Figure 5**). Gender composition could not moderate the relationship between materialistic values and subjective wellbeing.

**Table 6:** Publication year, percent female, and mean age as moderators (meta-regression).

				**95%** ***CI***			
**Moderators**	***k***	***estimate***	***SE***	***LL***	***UL***	***Z***	***Q _*model*_***
Materialistic values							
Publication year	40	−0.005	0.002	−0.008	−0.001	−2.601^**^	6.763^**^
% Female	36	0.001	0.001	−0.000	0.002	1.619	2.622
Mean age	21	0.010	0.001	0.008	0.013	7.622^***^	58.098^***^
Extrinsic aspirations							
Publication year	18	−0.011	0.003	−0.016	−0.006	−4.279 ^***^	18.348^***^
% Female	14	0.004	0.000	0.003	0.004	8.491^***^	72.104^***^
Mean age	12	0.009	0.002	0.005	0.012	5.182^***^	26.849^***^

Estimate, the regression coefficient of meta-regression; *Q*_model_, the *Q* value of meta-regression model; *LL*, lower limit; *UL*, upper limit;

** *p* < 0.01,

*** *p* < 0.001.

**Figure 4:**
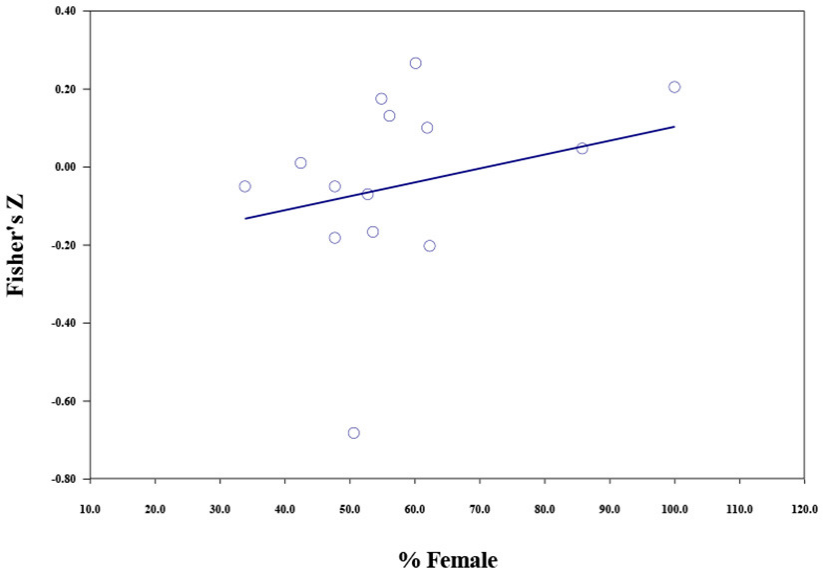
The moderating role of percent female in the association between extrinsic aspirations and subjective wellbeing.

**Figure 5:**
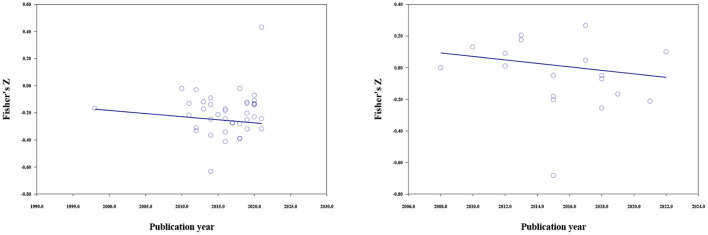
The moderating effect of publication year; materialistic values (left), extrinsic aspirations (right).

The relationship between extrinsic aspirations and subjective wellbeing was moderated by mean age (*b* = 0.009, *p* < 0.001), percent female (*b* = 0.003, *p* < 0.001), and publication year (*b* = −0.011, *p* < 0.001). The strength of the negative correlation increased with the publication year. And a larger proportion of female respondents and older participants in the samples predicted a weaker negative correlation (Figures 3–5).

#### Country economic indicators

**Figure 6:**
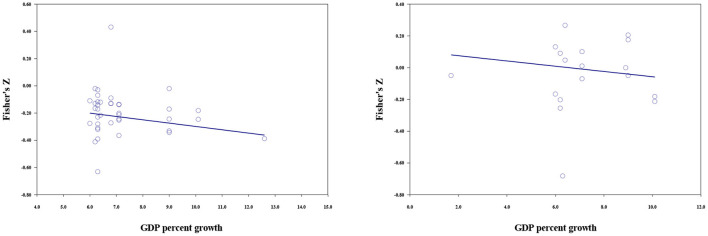
The moderating effect of GDP percent growth; materialistic values (left), extrinsic aspirations (right).

**Figure 7:**
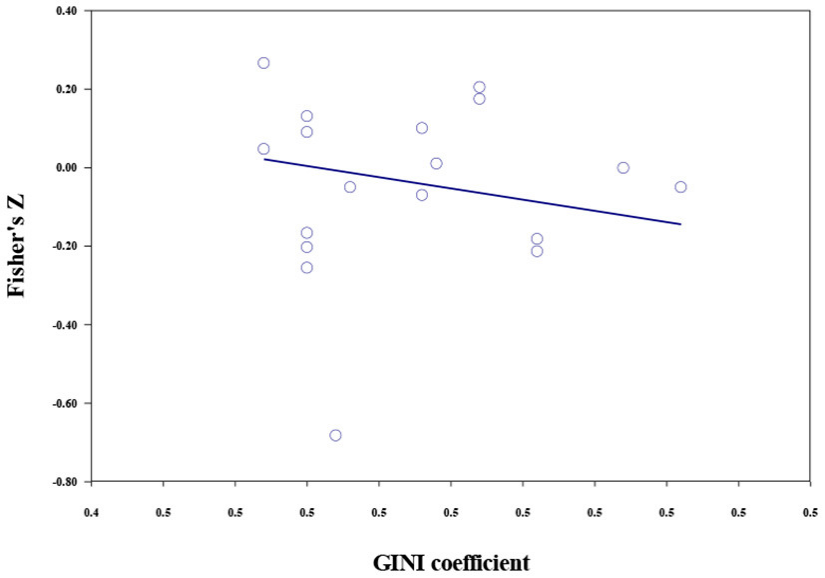
The moderating role of the GINI coefficient in the association between extrinsic aspirations and subjective wellbeing.

Meta-regression was used to examine the moderating effects of the economic indicators (per capita GDP indices, GDP percentage growth, and the GINI coefficient). The results of economic moderator analyses are shown in Table 7, Figures 6, 7. For materialistic values, although the moderation effect of GDP indices reached significance, the lower and upper limits were too close to 0, indicating that the effect was not robust. GDP percent growth could significantly moderate the size of the association between materialistic values and subjective wellbeing (*b* = −0.024, *p* < 0.001). The faster GDP grew, the stronger the negative correlation. The moderating effect of the GINI coefficient was not significant.

**Table 7:** Per capita GDP indices, GDP percent growth, and GINI coefficient as moderators (meta-regression).

				**95%** ***CI***			
**Moderators**	***k***	***estimate***	***SE***	***LL***	***UL***	***Z***	***Q _*model*_***
Materialistic values							
Per capital GDP indices	39	0.000	0.000	0.000	0.000	7.733^***^	59.806^***^
GDP percent growth	39	−0.024	0.003	−0.030	−0.019	−8.358^***^	69.850^***^
GINI coefficient	39	0.851	0.623	−0.301	2.073	1.365	1.864
Extrinsic aspirations							
Per capita GDP indices	18	0.000	0.000	−0.000	0.000	0.366	0.134
GDP percent growth	18	−0.017	0.004	−0.025	−0.008	−3.795 ^***^	14.404^***^
GINI coefficient	18	−5.710	0.829	−7.334	−4.085	−6.889 ^***^	47.453^***^

Estimate, the regression coefficient of meta-regression; the economic moderators are correlated and when all the three moderators are entered simultaneously in a multiple meta-regression, for materialistic values, the effect of per capita GDP indices no longer reaches significance (*b* = −0.000, *p* = 0.386), and the effect of GINI coefficient is still not significant (*b* = 0.676, *p* = 0.285), while the effect of GDP percent growth remains significant (*b* = −0.033, *p* = 0.0012). For extrinsic aspirations, although the effect of per capita GDP indices reaches significance (*b* = −0.000, *p* < 0.001), the lower and upper limits are too close to 0; the effect of GDP percent growth remains significant (*b* = −0.049, *p* < 0.001); the effect of GINI coefficient remains significance (*b* = −13.448, *p* < 0.001); *Q*_model_, the *Q* value of meta-regression model; *LL*, lower limit; *UL*, upper limit;

*** *p* < 0.001.

For extrinsic aspirations, the moderating effect of per capita GDP indices was not significant. GDP percent growth (*b* = −0.017, *p* < 0.001), and GINI coefficient (*b* = −5.710, *p* < 0.001) could moderate the relationship between extrinsic aspirations and subjective wellbeing. A higher GDP growth rate and GINI coefficient predicted a stronger negative correlation.

## Discussion

### Differential associations between two conceptualizations of materialism and subjective wellbeing

This meta-analysis examined how the two conceptualizations of materialism, materialistic values (Richins and Dawson, 1992) and extrinsic aspirations (Kasser and Ryan, 1993, 1996), relate to subjective wellbeing. The results showed the two conceptualizations of materialism to be markedly different. Materialistic values correlated negatively and significantly with subjective wellbeing: The mean effect size = −0.205, which is similar to prior meta-analyses (Dittmar et al., 2014; Zhou et al., 2018), supporting SDT. Conversely, there is no significant association between extrinsic aspirations and subjective wellbeing: The mean effect size = −0.048.

Materialistic values had a closer relation to lower subjective wellbeing than did extrinsic aspirations. This might be due to the fact that materialistic values make individuals more ego-involved in pursuing wealth and possessions. According to the conceptualization of Richins and Dawson (1992), materialistic values encourage individuals to relate their self-worth to wealth and possessions (e.g., the *success* dimension of MVS: defining one's success *via* wealth and possessions). Especially when someone is not fully financially satisfied, this may have a larger impact on self-concept (e.g., more negative self-evolution and lower self-esteem). And the ego-related problems can be more profoundly harmful to one's wellbeing. By contrast, extrinsic aspirations just manifest someone's tendency toward pursuing material success but do not directly reflect the level of ego involvement. Thus, pursuing wealth and possessions might not necessarily be psychologically problematic unless it is related closely to one's self-concept.

Another explanation for this finding is that the most widely used absolute measures of extrinsic aspirations in Chinese studies probably cannot capture the exact meaning of materialism. As mentioned above, the relative measures of extrinsic goals are more appropriate for materialism assessment because they locate the relative priority of materialistic goals within a broader goal system. Someone can be considered materialistic only when they prioritize materialistic goals over other goals. By contrast, the measure of materialistic values (Richins and Dawson, 1992) inherently captures the relative meaning of materialism (i.e., the *centrality* dimension measures whether wealth and possessions play a central role in one's life). Our results showed that relative scoring yielded a larger mean effect size of −0.190, close to that of materialistic values. Therefore, pursuing wealth and possessions might not harm people's wellbeing unless it is prioritized over other healthier intrinsic goals. And it could especially be the case in China in consideration of the dominant Cultural values in China, Confucianism, which emphasizes family/clan bonds and harmonious interpersonal relationships, which are close to the intrinsic goals (meaningful relationships) and may facilitate meeting the need for relatedness in the SDT context. Thus, the negative effects of extrinsic goals might be offset by the relatively high intrinsic goals among the Chinese (needs further investigations). Furthermore, the financial goals in China might also have an intrinsic meaning. In fact, because the Confucian culture places paramount importance on family (especially filial piety), pursuing financial goals in China is usually (at least partially) for filial purposes (i.e., providing better living conditions to parents and other elderly family members), which is closer to an intrinsic goal and perhaps counteracts the negative effects of the hedonic meaning of financial goals. This highlights the importance of using relative measures for extrinsic goals to control for intrinsic goals and may also indicate the need for an emic perspective in materialism research.

### Moderating factors

The negative correlations between materialistic values and subjective wellbeing were significant across different types of wellbeing outcomes. Most of the effect sizes were similar in size (ranging from −0.192 to −0.202), except for the significantly smaller one (−0.095) for positive affect. For extrinsic aspirations, the mean effect size for negative affect (reversed; −0.125) was the only significant one despite the non-significant difference among the wellbeing measures. Unexpectedly, the effect sizes for eudemonic wellbeing were not larger than those of the hedonic measures. Therefore, these results indicate that (1) there are differences between cognitive and affective aspects of subjective wellbeing in terms of the effects of materialism, and (2) there might be no essential difference in the effects of materialism on eudemonic and hedonic wellbeing.

The strength of the association between extrinsic aspirations and subjective wellbeing depended on scoring method of the AI. The effect size became significant and larger in the relative scoring subgroup (*r* = −0.190). As discussed above, the absolute measures of extrinsic aspirations used in most of the Chinese studies might account for the non-significant mean effect size of the correlation between extrinsic aspirations and subjective wellbeing. Relative measures were adopted too rarely in Chinese materialism literature. More empirical evidence provided by relative measures is needed to draw a more reliable conclusion.

Study design could moderate the association between extrinsic aspirations and subjective wellbeing. Longitudinal data yielded a significant and larger effect size (−0.254), indicating the potential lagged impact of extrinsic goals on wellbeing. This result also, to an extent, explains the non-significant association between extrinsic aspirations and subjective wellbeing, since most of the previous studies in China did not investigate the longitudinal effects of extrinsic aspirations.

The moderating effect of participant type was significant for extrinsic aspirations (but not materialistic values). For materialistic values, although there were no statistically significant differences in effect sizes across various types of participants, we observed a slightly stronger correlation in the youngest age group (elementary and/or secondary school students). This implies that the correlation might be affected by age.

The result for extrinsic aspirations also showed that there was even a significant and positive correlation in adults (*r* = 0.109). The result of the meta-regression showed that the correlations were less negative in older participants, indicating that materialism might be more problematic for adolescents. This might be because adolescents are more vulnerable to the discrepancy and identity deficits caused by materialism since they are at the crucial stage of the formation of identity and worldview (Zhou et al., 2018). This finding is the opposite of a prior cross-cultural meta-analysis (Dittmar et al., 2014) which suggests the internalization of materialism with age could be increasingly harmful.

The gender composition of samples could moderate the correlation between extrinsic aspirations (but not materialistic values) and subjective wellbeing. The negative correlation was weaker when women made up a greater proportion of the respondents. This might be because men are culturally expected to be dominant, aspirant, and power-oriented (see Eagly, 1997), which may lead to a higher level of internalization of extrinsic goals (e.g., status and financial success), thus suppressing other healthier intrinsic goals (e.g., caring relationships and helping others) which are usually connected to feminine traits. This may strengthen the negative effects of extrinsic goals on wellbeing among males. The possible reason why this moderation effect only occurred for extrinsic aspirations (but not materialistic values) might be that the absolute measures of extrinsic aspirations did not control for intrinsic goals. This finding once again highlights the importance of assessing relative material goals in materialism measurement.

Publication year (1998–2022) could moderate the relationship between materialism and subjective wellbeing. The size of the negative correlations increased over time. Since joining the WTO in 2001, Chinese society has experienced a decades-long surge in the market economy, advertising, and consumer culture (see Keane and Spurgeon, 2004). Given such an era background, the increasingly negative correlation can be attributed to the impact of consumer culture (Dittmar et al., 2013). That is, in the two-decade process of marketization and commercialization, people were more frequently exposed to consumerism messages *via* advertising, media, and their peers. According to the consumer culture values impact model (Dittmar et al., 2013), this social context will lead to a deeper internalization of materialism and stronger negative discrepancies, thus strengthening the negative effect of materialism on wellbeing (Dittmar et al., 2013, 2014; Zhou et al., 2018).

As noted above, the moderating effect of GDP was minimal, whereas GDP growth could moderate the associations. Materialism correlated closer to lower subjective wellbeing when the economy grew faster, thereby indicating that materialistic orientation could be more problematic for personal wellbeing in a booming economy. Faster GDP growth usually means more opportunities to access wealth (i.e., attain one's material goals). According to the logic of the goal-attainment perspective, the negative effect of materialism on wellbeing would have been weaker. Our finding does not support such a hypothesis but is consistent with the hypothesis derived from the consumer culture values impact model, which suggests that the more prosperous the economy, the greater the negative effects of materialism.

The correlation between extrinsic aspirations (but not materialistic values) and subjective wellbeing could be moderated by the GINI coefficient (wealth inequality). As predicted, greater wealth inequality could predict a larger negative correlation. Such a result can be attributed to the impacts of upward social comparison. The greater perceived wealth gaps may lead to more upward social comparison, thus causing more self-discrepancies and dissatisfaction among materialistic individuals.

### Implications, limitations, and future directions

The negative correlation between materialistic values (but not extrinsic aspirations) and subjective wellbeing among the Chinese found in this study is consistent with a prior cross-cultural meta-analysis (Dittmar et al., 2014), partially supporting the SDT account. This finding indicates that the effect of materialism might depend on whether it is defined as materialistic values or extrinsic goals, which could also be an open avenue for future research on the debate about whether materialism is necessarily harmful. As noted above, either the different extents of ego involvement or the absolute assessment (which cannot rule out the effect of intrinsic goals) could be the reason for the different results between materialistic values and extrinsic aspirations. Future research can further examine these two explanations.

Our results of the moderation effects of age, gender, economic growth, and wealth inequality are not consistent with or even contradict the findings of the cross-cultural meta-analysis (see Dittmar et al., 2014). This indicates that some global-level conclusions might be no longer valid within a given country. Given the relevance of materialism to culture, more research is needed to verify or diversify the validity of the global-level findings in a given nation.

Most of our findings on macro-level moderators (era and economic indicators) contradict the hypotheses based on the person-environment value congruence and goal attainment perspectives but support the consumer value impact model, suggesting that materialism may be more problematic for personal wellbeing in wealthier and more commercialized society (see Kasser et al., 2007; Dittmar et al., 2013). The consumer value impact model seems more valid in explaining the moderating effects of macro-level factors. Such a finding also suggests that interventions and policies aimed at reducing the endorsement of materialism might be more needed when the economy and consumer culture are booming.

Some limitations do exist in this study. First, because of the insufficient information provided by previous studies, we only examined country economic indicators rather than individual financial circumstances (e.g., income) in the moderation analyses. Thus, future research can further explore the potential moderating roles of individual financial circumstances or socioeconomic status in the relationship between materialism and subjective wellbeing. Second, there were few independent effect sizes for eudemonic wellbeing, relative scoring, and longitudinal data. The uneven distribution of the effect sizes in these subgroups may affect the robustness of the results of the subgroup analyses (Borenstein et al., 2009). Thus, future research can further examine the potentially differential effects of materialism on hedonic and eudemonic wellbeing. And, more studies using relative measures and longitudinal data are needed to advance materialism research in China.

## Conclusions

This meta-analysis demonstrates differential associations of materialistic values and extrinsic aspirations with subjective wellbeing. There is a significantly negative association between materialistic values and subjective wellbeing, while there is no significant correlation between extrinsic aspirations and subjective wellbeing. Thus, it is best that we do not rush to a conclusion on whether the materialism trend is generally harmful to the wellbeing among the Chinese. Instead, the effects of materialism might depend on individuals' ego involvement or levels of intrinsic goals as the two possible explanations we discussed above, which need to be examined more directly by future research.

The results of moderator analyses suggest that the negative association between materialistic values and subjective wellbeing is robust across most of the types of wellbeing outcomes, except for positive affect; the association between extrinsic aspirations and subjective wellbeing remains non-significant across different outcomes, except for negative affect. The association between materialistic values and subjective wellbeing is also moderated by type of publication, publication year, mean age, and GDP growth; the association between extrinsic aspirations and subjective wellbeing is moderated by type of participant, type of publication, scoring method of the AI, gender composition, publication year, mean age, GDP growth, and the GINI coefficient. These findings advance the literature by providing some evidence (from China) for the current debates (consumer culture value impact model vs. person-environment value congruence theory and goal-attainment perspective). In addition, the inconsistencies in the moderator results between the present research and a previous worldwide meta-analysis (Dittmar et al., 2014) highlight the importance of exploring the meaning and effects of materialism from an emic perspective.

## Data availability statement

The original contributions presented in the study are included in the article/Supplementary material, further inquiries can be directed to the corresponding author/s.

## Author contributions

KZ contributed to the study's conception and design. Material preparation and data collection were performed by LL and YW. Data analysis was performed by KZ. The first draft of the manuscript was written by KZ and LL. All authors commented on previous versions of the manuscript, read, and approved the final manuscript.

## Funding

This work was supported by the Political Science Research Funds of Sichuan Xiao Ping Executive Leadership Academy and the 2022 Systematic Scientific Research Project of Sichuan University of Socialism (No. XTKT202205).

## Conflict of interest

The authors declare that the research was conducted in the absence of any commercial or financial relationships that could be construed as a potential conflict of interest.

## Publisher's note

All claims expressed in this article are solely those of the authors and do not necessarily represent those of their affiliated organizations, or those of the publisher, the editors and the reviewers. Any product that may be evaluated in this article, or claim that may be made by its manufacturer, is not guaranteed or endorsed by the publisher.
